# Advances in Neural Engineering for Rehabilitation

**DOI:** 10.1155/2017/9240921

**Published:** 2017-12-20

**Authors:** Xiaoling Hu, Ting Zhao, Jun Yao, Yu Kuang, Yuan Yang

**Affiliations:** ^1^Interdisciplinary Division of Biomedical Engineering, The Hong Kong Polytechnic University, Hung Hom, Kowloon, Hong Kong; ^2^Howard Hughes Medical Institute, Janelia Research Campus, Ashburn, VA 20147, USA; ^3^Department of Physical Therapy and Human Movement Science, Feinberg School of Medicine, Northwestern University, 645 N Michigan Avenue, Suite 1100, Chicago, IL 60611, USA; ^4^Department of Medical Physics, University of Nevada, Las Vegas, NV 89154, USA

Neurorehabilitation has been identified as a grand challenge for the coming decades, mainly due to the fast-growing population with neurological disorders (e.g., stroke, Alzheimer's, and Parkinson's). Efficient, quantitative, and automated rehabilitation services are in urgent need to release the increasing demands for long-term medical treatments and healthcare and to compensate the lack of manpower in rehabilitation professionals. Neural engineering is an active research area, where engineering technologies, such as robots, imaging, biosignal processing, and sensors, have contributed to diagnosis, treatment, and long-term evaluation in rehabilitation processes. Advances in neural engineering techniques, from the fundamental research in laboratories to clinical trials, will definitely promote the automated and personalized rehabilitation in the future. There are ten articles collected in this special issue, featuring the cutting-edge representatives in the area of neural engineering.

A robot has been an important assistant to a human therapist in physical rehabilitation. The review articles, “Hand Rehabilitation Robotics on Poststroke Motor Recovery” by Z. Yue et al. and “Robotics in Lower-Limb Rehabilitation after Stroke” by X. Zhang et al., pointed out the therapeutic difficulties encountered in the traditional poststroke rehabilitation, that is, the recovery in distal joints and the restoration on walking independency. The papers summarized the latest developments in the robotic design and discussed the possible solutions to improve the performance of the current robots. In the article “Effects of Robot-Assisted Training for the Unaffected Arm in Patients with Hemiparetic Cerebral Palsy: A Proof-of-Concept Pilot Study” by A. Picelli et al., the positive rehabilitation effectiveness by practicing the unaffected upper limb with the assistance of robot has been validated, and the results demonstrated the improvements in hand functions and action planning ability in the recruited subjects. Rehabilitation robot was also applied in the study “The Effect of Dopaminergic Medication on Joint Kinematics during Haptic Movements in Individuals with Parkinson's Disease” by K. Li et al. The haptic sensitivity in individuals with Parkinson's disease, who received dopamine replacement therapy, was quantitatively evaluated in a robot-assisted haptic exploration.

Neural signal processing is a technology to understand the language talking in the nervous system. The neural signal of the brain detected by electroencephalography (EEG) was adopted as a biofeedback in the treatment for schizophrenia, as presented in “An Exploratory Study of Intensive Neurofeedback Training for Schizophrenia” by W. Nan et al. The study demonstrated the effectiveness of a short but intensive neurofeedback treatment for the patients with difficulty in long-time training and provided new insight into the treatment of schizophrenia. The neural signal of the brain was also investigated by electrocorticography (ECoG) in persons with epilepsy in the study “Gesture Decoding Using ECoG Signals from Human Sensorimotor Cortex: A Pilot Study” by Y. Li et al. The ECoG signals were used in a brain-machine interfacing (BMI) system to recognize different hand gestures performed by the subjects with an online accuracy above 80%. In the study “Prior Knowledge of Target Direction and Intended Movement Selection Improves Indirect Reaching Movement Decoding” by H. Li et al., the neural signals with higher resolutions than EEG and ECoG were captured by implanted microarrays at the cortical level in monkeys, and the neural signals were applied in the prediction of hand trajectories.

Quantitative evaluation plays an important role in diagnosis and long-term follow-up for rehabilitation. The imaging techniques of functional magnetic resonance imaging (fMRI) have been employed in the studies “The Difference of Neural Networks between Bimanual Antiphase and In-Phase Upper Limb Movements: A Preliminary Functional Magnetic Resonance Imaging Study” by Q. Lin et al. and “Cerebral Reorganization in Subacute Stroke Survivors after Virtual Reality-Based Training: A Preliminary Study” by X. Xiao et al. In Q. Lin et al.'s work, the effects of different bimanual practices in the upper limbs on the intra- and interregional connectivity in the brain were investigated in unimpaired subjects, and the results revealed the behavioral modulation on the cerebellar-cerebral functional connectivity. In X. Xiao et al.'s work, fMRI imaging was applied in the evaluation on the poststroke rehabilitation program by virtual reality-enhanced treadmill training. The neural reconstruction in the primary sensorimotor cortex after the training could be determined with the imaging quantification. In the study “Characterizing Patients with Unilateral Vestibular Hypofunction Using Kinematic Variability and Local Dynamic Stability during Treadmill Walking” by P. Liu et al., the asymmetry and instability during the gait of the patients were evaluated by three-dimensional motion analysis. The severity of vestibular functional asymmetry could be quantified by the parameters of the motion analysis on the lower limbs, which could be complementary to the traditional assessments.

We hope that this special issue of Behavioral Neurology will help to promote further developments in neural engineering and neurorehabilitation. In addition to reducing suffering and improving the quality of life, neurorehabilitation when combined with novel engineering methods has the potential to advance our knowledge about the mechanisms of the nervous system.

